# Treatment Adherence Interventions for Burn Patients: What Works and What Role Can Motivational Interviewing Play?

**DOI:** 10.3390/ebj3020026

**Published:** 2022-04-06

**Authors:** Christian R. R. Goans, Karen J. Meltzer, Blake Martin, Kimberly Roaten

**Affiliations:** UT Southwestern Medical Center, Dallas, TX 75390, USA; karen.meltzer@utsouthwestern.edu (K.J.M.); blake.martin@utsouthwestern.edu (B.M.); kimberly.roaten@utsouthwestern.edu (K.R.)

**Keywords:** treatment adherence, motivational interviewing, intervention, psychology, interdisciplinary

## Abstract

The unique challenges burn patients face along the trajectory of recovery necessitate an interdisciplinary team approach to care. As much as providers rely on care-team members for delivery of optimal treatment, the patient must be an active collaborator in their care. Optimal burn recovery outcomes hinge on treatment adherence. In addition to general challenges faced in ubiquity by burn patients, there are specific patient populations for whom treatment adherence is particularly challenging. Although psychological interventions have been used successfully with burn patients, very few are appropriate for both inpatient and outpatient care environments and most do not focus on treatment adherence. This paper reviews unique facets of Motivational Interviewing (MI) that may be applicable in interdisciplinary burn treatment teams across inpatient and outpatient settings to optimize treatment adherence.

## 1. Introduction

Optimal burn treatment requires active collaboration from the patient in the form of adherence, defined as the extent to which recommended treatments are implemented by the patient [1]. Psychological interventions for treatment adherence among burn survivors are primarily focused on inpatient treatment, whereas comparatively little data exist on outpatient treatment, such as follow-up appointment attendance, home-based dressing changes, and at-home range-of-motion exercises [2]. This article will begin with a review of motivational interviewing (MI), cover challenges burn patients encounter, and describe validated psychological interventions before discussing specific barriers to treatment adherence with several potential applications of MI interventions and suggestions for future research.

## 2. Motivational Interviewing

After its initial development as a treatment for alcohol abuse [3], MI has been applied across a wide variety of behavior change domains. The most recent edition of the MI handbook cited over 1200 publications with the rate of publication doubling every three years since 1990 [4]. The manual defines MI as “a collaborative, goal-oriented style of communication with particular attention to the language of change. It is designed to strengthen personal motivation for and commitment to a specific goal by eliciting and exploring the person’s own reasons for change within an atmosphere of acceptance and compassion” [4] (p. 29). The development of MI coincided with seminal research on the Transtheoretical Model (TTM) [5,6], out of which arose the stages of change. MI interventions focus on the contemplative stage of change, during which the patient experiences ambivalence characterized by motivation both for and against behavior change. In a conversation about behavior change, patients will talk about changing (“change talk”) along with keeping things as they are (“sustain talk”).

The natural reaction for providers when a patient speaks about maintaining a problematic pattern of behavior is to argue for change; unfortunately, this natural reaction fails. Termed “the righting reflex” in MI literature, attempts to convince the patient to change further entrench the patient in their harmful behavior patterns via reactance. The solution to this “righting reflex” problem is to respond, rather than react, when a patient speaks against behavior change. A more helpful provider response is to guide the patient’s exploration of their own ambivalence.

MI’s solution to the patient’s sustain talk and the provider’s desire to fix it has elements of both *process* and *content*. The *process* element is the “Spirit of MI”, which is broken down into four key components: collaboration, acceptance, evocation, and compassion [4,7]. These four principles undergird any specific MI technique used with a patient. The foundational *content* of MI is a core set of skills represented with the acronym OARS: open-ended questions, affirmations, reflections, and summaries [8]. The purpose of closed-ended question is rapid assessment, whereas the purpose of open-ended questions is to elicit elaboration. Affirmations, such as praising the patient for talking openly with the provider, also propel the conversation toward change. Reflections have multiple levels of complexity, but in essence, re-state the patient’s words back to them to expand on statements about behavior change. Adding reflections is more likely to yield an elaborative conversation about change compared to back-and-forth volleys of provider questions and patient answers. These four specific skills are designed to drive the patient’s momentum toward more and more change talk.

MI techniques, including OARS and others, aim to encourage the patient to talk more and more about changing their behavior. Research on MI treatment fidelity and clinical outcomes strongly supports that change talk is the primary indicator of behavior change [9]. The reason for this is simple: talking about change predicts actual change in behavior [10,11].

MI has empirical support as a treatment adherence intervention in populations with chronic disease that face psychosocial challenges similar to the burn population, such as adults with chronic pain [12], who share with burn patients the need for potentially painful physical activity to promote healing and range of motion. MI interventions are also shown to improve treatment adherence in patients with chronic kidney disease, who often face disability and need time-consuming hemodialysis [13] in addition to those with HIV, who must attend regular medical appointments, have strict medication adherence, and are at a high risk for multiple psychosocial stressors (e.g., psychiatric problems, social and vocational stigma, substance use problems) [14]. Randomized controlled trials (RCTs) have demonstrated that MI promotes adherence to lifestyle interventions for obesity [15], exercise programs for fall prevention [16], CPAP [17], and physical therapy post-stroke [18]. Similarly, RCTs yielded positive results for MI’s effect on adherence to statins [19], immunosuppressants after transplantation [20], insulin [21], and antiretrovirals [22]. There is strong evidence for the use of MI to promote adherence in the above domains, but there is a lack of research on MI for treatment adherence across the complex spectrum of burn treatment.

## 3. Challenges during Recovery from Burn Injury

As mortality following a burn has decreased due to improvements in care, there has been an increased focus on the psychological needs of burn survivors, with an emphasis on reducing distress and promoting long-term well-being [23]. Psychological factors have a significant influence on the course of recovery following a burn. For example, burn patients with pre-existing mental health conditions have longer stays in burn treatment inpatient units and undergo more surgeries while inpatient [24,25]. Following discharge, the psychological effects of the burn may continue to impact patients’ functioning, impeding the return to work or school, leading to increased social isolation, and negatively impacting overall adjustment [26,27].

The majority of burn patients report distress and can benefit from some form of psychological care [28]. While burn survivors are a heterogeneous group, patients with burns face a myriad of shared challenges during recovery, and the nature of these challenges is often related to the patient’s stage of recovery [29]. These stages are multifactorial and vary in duration [30]. We will briefly review the common psychological challenges that may be encountered during the stages of physiological recovery: the resuscitative/critical stage, the acute stage, and the long-term rehabilitative stage [30].

### 3.1. Resuscitative/Critical Stage

The Resuscitative/Critical Stage is characterized by the patient’s fight for survival and may occur in an intensive or acute care unit. At this stage, major stressors include uncertainty about recovery, pain, and stressors from a prolonged intensive care stay [29,31]. Delirium is common during this phase, affecting 30% to 70% of burn patients within 48 h of burn injury [32]. Furthermore, intubation during this stage may impair communication between the patient and the care team [33].

### 3.2. Acute Stage

The acute stage often consists of painful restorative care that can extend to the outpatient setting. Patients become more alert and oriented during this stage, with increasing awareness of pain and less sedation during wound care and rehabilitative therapies [31,34]. Additionally, patients may begin comprehending the extent of their physical injuries as well as the psychological impact of these injuries [29]. Challenges faced during this stage of recovery include depressive, anxious, and traumatic stress symptoms, disturbances in sleep, grief/bereavement, and intensification of premorbid psychopathology. We will briefly review each of these challenges to give context for the implementation of strategies to improve treatment adherence across the full spectrum of recovery.

#### 3.2.1. Depression, Anxiety, and Traumatic Stress

Depressive and anxious symptoms are quite common following burn injury during the acute stage of recovery. Approximately 22 to 54% of burn survivors exhibit at least mild depressive symptoms, and 13 to 26% display moderate to severe depressive symptoms [35]. Anxiety disorders are also common, with over 21% of burn survivors presenting with an anxiety disorder six months after the burn [36].

Furthermore, trauma-related symptoms may surface in the form of Acute Stress Disorder (ASD; occurring in the first month after the burn) and post-traumatic stress disorder (PTSD; occurring after one month) [37]. PTSD affects nearly one-half of burn survivors, with burn centers reporting prevalence rates ranging from 8 to 45% [38,39,40]. Even one year after the burn, up to 45% of adults who were hospitalized for their burn injury meet criteria for PTSD [41,42].

#### 3.2.2. Sleep Disturbance

Sleep disturbance is a common symptom of anxiety, depressive, and trauma-related disorders. However, the relationship between sleep and these disorders is bidirectional [43,44,45]. Along with the psychological factors leading to sleep disturbance, the hospital environment itself often contributes to poor sleep [46]. Loud noises, repeated nighttime interactions with nursing staff, as well as disruptions in circadian rhythm may all contribute to sleep disturbance [47].

#### 3.2.3. Grief

Grief is common as patients begin to reckon with the impact of the burn injury on their lives [48]. The event that caused the injury may have also led to tragic outcomes such as the death or serious injury of loved ones or pets, the loss of property or jobs, and the loss of mobility, ability, and appearance [29].

### 3.3. Long-Term Rehabilitative Stage

This stage of recovery often begins during inpatient rehabilitation and continues beyond discharge from the hospital as patients reintegrate into the community [49]. Higher burn severity corresponds to higher frequency of rehabilitative therapies, dressing changes, and surgeries. The first year after discharge from the hospital has been found to be a period of especially high distress [29].

During this period, patients may face both physical and psychosocial challenges. In the physical domain, patients may face a variety of frustrations such as decreased endurance, difficulty with dexterity, and pruritis. Pruritus affects over 90% of burn patients and persists long-term in over 40% [50]. Further complications, such as amputation, neuropathic pain, scarring, and heterotopic ossification, can arise at this stage [29].

Psychosocially, patients may face an array of stressors, including return to work, disability applications, relational strain, changes in sexual functioning, altered body image, and disruptions in activities of daily living. Financial concerns may be especially salient among burn survivors for the first two years following discharge [51,52,53]. Specifically, while physical barriers may initially impact the ability to work, psychosocial factors such as nightmares and self-image concerns may have a greater impact on the ability to work long-term [26]. The passage of time itself improves patients’ adjustment to their injury regardless of burn severity, and social support significantly moderates the psychological impact of the injury in the long term [54,55]. While some individuals may naturally recover over time, others may need formal psychological intervention during this period.

## 4. Existing Psychological Interventions for Burn Survivors

Proper burn care is critical to first ensure survival and ultimately promote positive health outcomes [2]. In their systematic review of treatment adherence among burn survivors, Szabo et al. (2016) noted four treatment areas where burn researchers have focused on adherence: (1) diet, (2) pressure garment therapy/silicone gel sheeting, (3) PT/OT/exercise, and (4) first aid/follow-up care [2]. Generally, the authors found little data on adherence to follow-up appointments, range-of-motion exercises, dietary regimens, and home-based dressing changes. Overall, the review found educational and behavioral interventions to be promising in improving treatment adherence across all stages of burn treatment. The literature on medical treatment adherence in general suggests a link between social support and better adherence [56]. See Table 1 for a summary of existing psychological interventions for treatment adherence among burn patients.

### 4.1. Interventions for Psychological Factors Affecting Treatment Adherence

Past research has demonstrated that burn survivors rated as lower in adherence by physiotherapists had longer LOS than patients with higher adherence ratings, and patients rated poor in adherence were more likely to have a psychiatric diagnosis [25]. Weichman and Patterson (2004) emphasized the importance of considering patients’ psychological functioning throughout all stages of burn injury: critical care, acute care, and rehabilitation [29]. The authors noted that initial emphasis on survival in the resuscitative/critical stage of care may overshadow psychological factors that complicate long-term recovery. However, patients may begin working with mental health professionals during the acute phase, when virtual reality, hypnosis, guided imagery, and relaxation techniques can facilitate coping with painful procedures and adjustment to a long hospital stay. Virtual reality may be effective during important ROM exercises in the rehabilitation phase [58]. Patients may also be experiencing grief, guilt, or bereavement; trauma-informed psychotherapy can help patients process complex emotions which are common barriers to adherence.

Procedural pain and background pain are distressing for patients and often difficult to treat with pharmacotherapy alone. Viewed through an operant conditioning lens, medication administration becomes associated with exhibiting pain behaviors in a negative reinforcement loop, wherein continuing the pain behaviors results in reduction of pain through medication. Provision or restriction of pain medication alone cannot break this reinforcement cycle vaccines-1643738. Classical conditioning principles are also helpful for understanding and treating distress in burn rehabilitation [60]. For example, patients may develop anxiety or problems sleeping if painful procedures are always conducted in their hospital room. Mental health clinicians may note these associations and work collaboratively with the team on a plan to conduct certain procedures away from the patient’s room to weaken the distressing association. Cognitive behavior therapy (CBT) incorporates the conditioning principles with tracking and restructuring pain-related thoughts in order to reduce pain and improve adherence [29].

Askay et al. (2009) noted that assessing a patient’s coping style can lead to better outcomes in burn treatment adherence, underscoring the importance of understanding individual traits [60]. Patients with an approach coping style may appreciate collaboratively setting weekly goals with their physical therapist and reading articles about certain treatments, while an avoidant patient may prefer their therapist to set goals and have a family member present to provide distracting conversation during a difficult dressing change.

### 4.2. Interventions for Rehabilitative Therapies and Exercise

Few studies have examined adherence to PT/OT and exercise regimens in burn survivors. Numerous techniques are utilized to improve treatment adherence in both adults and children: regular scheduling, relaxation techniques, scare tactics, breaks, rewards, contracting, range-of-motion boards, buddy systems, predictable schedules, and relaxation techniques [63]. The same study found range-of-motion and stretching exercises associated with the lowest adherence, while activities of daily living (ADLs) were associated with the highest adherence. Another study found better adherence to PT when patients were given the option of self-directed exercise [64]. Psychological factors have also been implicated in PT/OT adherence. Patients rated by providers as higher in self-blame about their burn injuries were rated by staff as lower in PT/OT adherence [65]. These results reflect the importance of psychotherapy as an intervention to address negative cognitions and emotions, which may result in improved adherence to PT/OT.

An additional targeted intervention for adherence to PT/OT is the quota system [66]. Once a target behavior is established, such as sitting up or walking, the patient and provider establish a baseline level of performance over three to five days. The provider calculates average performance over the baseline period, then the new daily target is set for 50 to 80% of the baseline average. The daily target increases by 5 to 10% each day. The quota system increases the patient’s sense of control and mastery within the burn care environment, where limited perception of control over outcomes can lead to helplessness and ultimately diminish adherence.

### 4.3. Interventions for Pressure Garment Adherence

Pressure garment therapy is often used for prevention of hypertrophic scarring [67]. Primary barriers to adherence include physical complaints, such as pain, itching, perspiration, blistering, ulceration, and rashes, in addition to shame and embarrassment associated with wearing the garments in public [61]. Several factors have been associated with better pressure garment adherence: the opportunity to meet with other burn survivors, seeing photographs of outcomes, having color options, social support, personal factors, and beliefs in the efficacy of treatment, with understanding the benefits and physical characteristics (fit and color options) of the pressure garments being the strongest predictors [2]. Researchers recommend providers take extra time to ensure patients understand the benefits of and expected challenges to wearing pressure garments. Psychoeducation regarding common emotional reactions to wearing the garments can help prepare patients for future barriers and plan for adaptive coping strategies. Assertiveness and other communication skills training can help patients prepare for social situations in which they may encounter questions about their garments [61]. Ripper et al. (2009) recommended small group education interventions to address these concerns [61].

### 4.4. Interventions for Outpatient Adherence

Few studies have examined adherence to outpatient treatment after burn injury. A recent retrospective chart review study found more than 30% of patients did not follow-up at one week, almost 75% at three months, and more than 90% at six months [68]. The same study found that being ≤12 years of age, having ≥1 operation during inpatient treatment, and residing closer to the hospital were all associated with higher likelihood of outpatient follow-up.

Psychological factors pose barriers to management of burn wounds after hospitalization. Patients with untreated psychotic disorders, for example, are likely to face barriers to adherence such as inability to manage dressings and even homelessness [69]. The bidirectional relationship between higher depression and anxiety scores and higher levels of pain, fatigue, and worse physical functioning up to two years post-discharge makes the involvement of mental health clinicians equally important during inpatient treatment and following discharge [70]. Psychotherapy during inpatient care could enhance chronic medical and psychiatric illness treatment adherence after discharge (e.g., medication, diet, exercise, and doctor’s visits). Interventions for substance use disorders are essential both during inpatient and outpatient treatment in order to address these barriers to adherence.

## 5. Barriers to Treatment Adherence among Burn Patients and Potential MI Applications

### 5.1. Patient Factors

The high prevalence of psychiatric illness among burn patients is a potential barrier to treatment adherence. Patients with psychotic and/or substance use disorders are less likely to engage in adaptive coping or treatment adherence [71]. Researchers have found drug and/or alcohol intoxication in at least half of patients admitted for acute burn [62,72,73]. Palmu et al. (2018) also found patients who were intoxicated at the time of their injury had a higher prevalence of lifetime psychiatric disorders compared to those who were not intoxicated, with alcohol use disorder and anxiety disorders being the most common [62]. Alcohol use disorder and psychotic disorders were most common in individuals who were both smoking and under the influence of alcohol at the time of burn injury. A positive drug screen was noted in more than half of burn patients in one study, with opiates being the most commonly reported, followed by stimulants and marijuana [72]. At the time of injury, almost one-fifth of burn survivors in one study reported positive scores for a self-report measure of substance use problems called the CAGE (Cut-down, Annoyed, Guilty, and Eye-opener) [73]. More than half of those participants reported positive scores at a follow-up time point, suggesting substance use problems persist beyond injury. Additionally, the rate of patients testing positive for cannabis is increasing rapidly, likely due to more states legalizing marijuana [59].

Given the high prevalence of substance use among burn injury survivors, MI interventions to increase overall treatment adherence with concurrent substance recovery may be effective. Fortunately, MI principles, developed initially for alcohol cessation, can address ways to maximize reinforcement (affirmation), while minimizing punishment (manifestation of the righting reflex). For example, most patients are already aware of the dangers of nicotine use, whether or not they understand that nicotine complicates wound healing, but patients are far less likely to know about the health benefits of cessation and how quickly the body can recover from nicotine use. This kind of education, focused on positives to be gained through cessation rather than negatives to be avoided, is less likely to elicit reactance and more likely to appeal to the patient’s inherent desire to make a change in their tobacco/nicotine use.

### 5.2. Psychological Factors and Inpatient Complications

Both pre-existing and in-hospital development of psychiatric illness have been linked to worse clinical outcomes in acute burn care, including higher number of procedures, elevated infection risk, longer length of stay (LOS), and poorer treatment adherence [8,25,69,73]. With so many complex factors and treatments involved, continual patient education is an important component of inpatient burn treatment. By optimizing how we inform patients about next steps in care, providers can maximize the likelihood that patients will be adherent to treatment across the course of their recovery. MI offers some concrete principles for effective education/informing [74]. At the foundation, requesting the patient’s permission before informing about a given topic honors the patient’s autonomy and increases the likelihood that the patient will be invested in learning the information provided.

An additional element of informing the patient is to frame education in terms of what others in similar positions do to maximize quality of care. For example, if the patient has not been adherent with rehabilitative therapies, the righting reflex might lead a provider to directly inform the patient of the risks of physical inactivity. This approach is likely to elicit reactance from the patient and is unlikely to increase adherence. Conversely, if the provider frames information in terms of what others do, they can provide the same information, while minimizing the risk of reactance as a barrier to adherence. For example, “many patients struggle with the physical exertion that PT requires, but those who choose to trust the therapists to know where the limits are and push as hard as possible every day see the best results in their overall treatment”.

Many providers elicit feedback from patients while informing by checking for understanding as they move through material. MI offers a way to further optimize this approach by preempting this back-and-forth with a question about the patient’s current knowledge of a particular education domain. This initial step can simultaneously honor the patient’s autonomy, demonstrate respectful collaboration, and gain the patient’s permission all in one simple open-ended question: “What do you know about X”? In the MI literature, this technique is termed elicit-provide-elicit [74]. First, elicit the patient’s knowledge about a given topic. Second, provide information to the patient. Third, elicit the patient’s takeaways from the provided information.

### 5.3. Psychological Factors and Outpatient Complications

Psychiatric problems are associated with poor long-term outcomes after burn injury, such as worsened pain and fatigue, lower physical functioning, increased need for home health services at discharge, and poorer health-related quality of life (HRQOL) [70,73]. Long-term burn rehabilitation in the outpatient setting involves frequent collaboration between patients and medical teams to set and adjust treatment goals. The second core process in MI following initial engagement with the patient, is focusing on a target behavior for change. The provider, as the treatment expert, knows what treatment elements are necessary to advance the trajectory of recovery. However, the patient must be an active participant in goal setting. For an example, the quota system clearly defines its primary purpose as an intervention for learned helplessness that results in increased adherence to rehabilitative therapies as well as pressure garments. By setting conservative goals for the target behavior and making sustainable, incremental increases with progress, the patient is empowered to continue increasing flexibility, strength, and healing overall. However, the quota system begins with the assumption that the target behavior has already been selected. How much more powerful could this process be if the patient was first empowered to select a target behavior from a curated set of options?

Offering choices allows providers to honor the patient’s autonomy and increase adherence. These choices should be offered simultaneously with careful curation from the provider as the expert on treatment [74]. This technique may be applied more broadly than the previous work regarding pressure garment adherence [2]. Rehabilitation therapists can offer the patient choices, where feasible, via a list of tasks for a given session. For example, if range-of-motion exercises are indicated for multiple limbs, the patient could sequence those exercises according to their preference. Wound dressing changes can apply the same logic, allowing the patient to choose which body areas are addressed first. This small element of curated choice can reduce patient reactance by supporting patient autonomy.

This idea of curation can expand to the application of core MI skills as well. Providers can guide the conversation toward change talk with open-ended questions followed by targeted reflections. For a patient who engages variably in PT, asking: “What has been helping you get through your PT sessions”? is likely to elicit change talk, such as reasons for PT engagement in order to return to work as soon as possible, desire to continue playing actively with grandchildren, or consideration of underlying resilience evident in a patient’s prior response to adversity. If the provider then reflects the patient’s reasons, desire, or perceived ability for adherence, the patient is likely to continue expanding on those elements of the conversation, all of which are rich examples of change talk.

## 6. Conclusions

This review summarizes many of the challenges burn survivors may face as they recover from their injuries, with a specific focus on barriers to treatment adherence. Evidence-based psychological interventions are summarized, and MI is introduced as a powerful intervention that may be particularly useful for addressing adherence issues across a variety of clinical settings throughout the phases of healing. MI need not compete with the existing, robust treatment approaches described above. Rather, principles inherent to the MI spirit and OARS techniques can be woven throughout patient interactions across treatment stages.

For optimal MI fidelity, psychologists or other mental health professionals can seek out the motivational interviewing network of trainers (MINT) [75]. Allied interdisciplinary burn treatment team members can also utilize MI principles in their work with patients. The consistent application of the MI spirit, along with MI techniques during patient interactions, is likely to decrease reactance on the part of the patients while simultaneously connecting the patient with core values that will promote treatment adherence and holistic, values-congruent lifestyle choices. It is our hope that this review encourages creative implementation of MI-informed practices as well as robust scientific evaluation of the efficacy of MI to improve treatment adherence and other clinical outcomes among burn survivors.

## Figures and Tables

**Table 1:** Existing psychological interventions for burn treatment adherence.

Intervention	Benefits	Limitations	Relevant Citations
Behavioral interventions for distraction (e.g., hypnosis, virtual reality, guided imagery relaxation training)	Improved pain control Useful to build rapport in acute care	VR requires equipment; hypnosis requires specialized training	Patterson et al., 2021 [57] Soltani et al., 2018 [58] Weichman & Patterson, 2004 [29]
Bereavement and trauma-informed psychotherapy	Considers patient’s stage of emotional processing of injuries and losses May facilitate subsequent participation in treatment	Does not directly promote behavior change	Weichman & Patterson, 2004 [59]
Cognitive Behavioral Therapy (CBT)	Improves pain control, lessens distress, and reduces maladaptive pain behaviors	Requires specific provider training Poor delivery of CBT can fail to build rapport	Askay et al., 2009 [60]
Assessment of patient’s coping style	Supports patients’ active role in their care Educates interdisciplinary team members to improve treatment delivery	Primarily used in long-term rehabilitative stage with limited acute stage applications	Askay et al., 2009 [10]
Psychoeducation	Prepares patients for upcoming challenges	Not comprehensive Requires additional skills and specific coping techniques	Ripper et al., 2009 [61]
Assertiveness and communication skills training	Improves communication between patients, providers, and supporters to improve quality of care and support Facilitates faster re-integration into social lives and improve long-term adjustment	Not comprehensive Most helpful in group settings which are not always available	Ripper et al., 2009 [61]
Substance use treatment	May help with appointment follow-up and home wound care May prevent future injuries	High attrition rates	Palmu et al., 2018 [62]
